# The Course of Skin and Serum Biomarkers of Advanced Glycation Endproducts and Its Association with Oxidative Stress, Inflammation, Disease Severity, and Mortality during ICU Admission in Critically Ill Patients: Results from a Prospective Pilot Study

**DOI:** 10.1371/journal.pone.0160893

**Published:** 2016-08-16

**Authors:** John H. Meertens, Hans L. Nienhuis, Joop D. Lefrandt, Casper G. Schalkwijk, Kristiina Nyyssönen, Jack J. M. Ligtenberg, Andries J. Smit, Jan G. Zijlstra, D. J. Mulder

**Affiliations:** 1 Department of Critical Care, University Medical Center Groningen, University of Groningen, Groningen, The Netherlands; 2 Department of Internal Medicine, University Medical Center Groningen, University of Groningen, Groningen, The Netherlands; 3 Laboratory for Metabolism and Vascular Medicine, Department of Internal Medicine, Maastricht University Medical Center, Maastricht, The Netherlands; 4 Institute of Public Health and Clinical Nutrition, Department of Clinical Chemistry, University of Eastern Finland, Kuopio, Finland; 5 Eastern Finland Laboratory Center, Kuopio, Finland; University of Colorado Denver School of Medicine, UNITED STATES

## Abstract

**Background:**

Advanced glycation end products (AGEs) have been implicated in multiple organ failure, predominantly via their cellular receptor (RAGE) in preclinical studies. Little is known about the time course and prognostic relevance of AGEs in critically ill human patients, including those with severe sepsis.

**Objective:**

1) To explore the reliability of Skin Autofluorescence (AF) as an index of tissue AGEs in ICU patients, 2) to compare its levels to healthy controls, 3) to describe the time course of AGEs and influencing factors during ICU admission, and 4) to explore their association with disease severity, outcome, and markers of oxidative stress and inflammation.

**Methods:**

Skin AF, serum N"-(carboxyethyl)lysine (CEL), N"-(carboxymethyl)lysine (CML), and soluble RAGE (sRAGE) were serially measured for a maximum of 7 days in critically ill ICU patients with multiple organ failure and compared to age-matched healthy controls. Correlations with (changes in) clinical parameters of disease severity, LDL dienes, and CRP were studied and survival analysis for in-hospital mortality was performed.

**Results:**

Forty-five ICU patients (age: 59±15 years; 60% male), and 37 healthy controls (59±14; 68%) were included. Skin AF measurements in ICU patients were reproducible (CV right-left arm: 13%, day-to-day: 10%), with confounding effects of skin reflectance and plasma bilirubin levels. Skin AF was higher in ICU patients vs healthy controls (2.7±0.7 vs 1.8±0.3 au; p<0.001). Serum CEL (23±10 vs, 16±3 nmol/gr protein; p<0.001), LDL dienes (19 (15–23) vs. 9 (8–11) μmol/mmol cholesterol; <0.001), and sRAGE (1547 (998–2496) vs. 1042 (824–1388) pg/ml; p = 0.003) were significantly higher in ICU patients compared to healthy controls, while CML was not different (27 (20–39) vs 29 (25–33) nmol/gr protein). While CRP and LDL dienes decreased significantly, Skin AF and serum AGEs and sRAGE did not change significantly during the first 7 days of ICU admission. CML and CEL were strongly correlated with SOFA scores and CML above the median at baseline was associated with increased risk for mortality (Hazard ratio 3.3 (1.3–8.3); p = 0.01). All other markers did not correlate with disease severity and did not predict mortality.

**Conclusions:**

This study demonstrates that markers for the AGE-RAGE axis are elevated in critically ill patients compared to healthy controls but remain stable for at least 7 days despite clearly fading inflammation and oxidative stress. Circulating AGEs may be associated with disease severity and outcome. Further research should be conducted to elucidate the role of the AGE-RAGE axis in the exaggerated inflammatory response leading to multiple organ failure and death, and whether or not this may be a target for treatment.

## Introduction

Multiple organ failure is often caused by the systemic inflammatory response syndrome and is associated with high mortality [1,2]. Although the systemic inflammatory response syndrome is a physiologic host response to infection and injury, it is also accompanied by the production of reactive oxygen species and insufficiency of the detoxifying system, leading to oxidative stress [3]. Oxidative stress has been shown to promote the development of multiple organ failure [4] and is associated with an unfavourable outcome in patients with sepsis [5].

Unfortunately, assessment of oxidative stress in clinical studies is difficult because of the highly unstable nature of reactive oxygen species [6]. Advanced glycation end products (AGEs) are stable products, originally only considered as products of the slowly occurring non-enzymatic glycation (Maillard reaction) in chronic diseases such as diabetes. However, AGEs are also rapidly formed during oxidative stress, which implicates a potential role in sepsis [7–12]. In preclinical studies, AGEs have been shown to exaggerate the inflammatory response. They engage the multiligand receptor for AGEs (RAGE) [13] to boost septicaemia [14]. RAGE-deficient mice are strongly protected against mortality due to polymicrobial sepsis [15]. Indeed, in human sepsis studies, skin autofluorescence (Skin AF), a validated non-invasive tissue marker for cross linking AGEs [16], and antibodies against non-N"-(carboxymethyl)lysine (CML) AGEs [17] were significantly elevated. However, these markers for AGEs are not exclusively derived from oxidative stress.

We performed an explorative pilot study in which we hypothesized that in critically ill patients both tissue (Skin AF) and circulating AGEs (CML and N"-(carboxyethyl)lysine (CEL)) are elevated in concordance with the soluble receptor for AGEs and markers of lipid peroxidative stress (LDL dienes) and inflammation (CRP) and that particularly circulating AGEs are associated with disease severity and outcome. For this purpose, these markers were serially measured in a well-defined prospective cohort of critically ill patients admitted to our ICU and compared to healthy controls.

## Methods

### Subjects

#### Critically ill patients

Consecutive patients admitted to a 12 bed mixed medical/surgical intensive care unit of the University Medical Center Groningen were screened for eligibility. To guarantee critical illness, the inclusion criterion was an Apache II score of more than 18 points in a 24 h-period immediately before inclusion at an arbitrary moment during ICU admission. Exclusion criteria were age less than 18 years and a history of diabetes or renal disease (serum creatinine >150 μmol/l before current admission), the latter being conditions classically associated with chronically increased AGE accumulation. Skin AF was measured and blood samples were collected daily from inclusion (day 1) to day 7. Clinical data were prospectively obtained. The study has been conducted according to the principles expressed in the Declaration of Helsinki and was approved by the Institutional Review Board of the University Medical Center Groningen, University of Groningen, The Netherlands (METc 2005/007). Each participant or next of kin gave written informed consent. Medical treatment was according to physicians’ discretion. As standard practice at our ICU, blood glucose levels were regulated by short acting insulin infusion, aiming at glucose levels between 5.0 and 7.0 mmol/L [18], and patients were fed via an enteral tube at a constant rate 24 hours a day.

#### Control subjects: healthy controls

A group of healthy controls was included for comparison. This was an age-matched historical control group of 37 volunteers, who were recruited by advertisement and did not have more than one of the following cardiovascular risk factors: hypertension, smoking, hypercholesterolemia or a family history of cardiovascular disease. They also had normal femoral and carotid arteries on ultrasound examination. Exclusion criteria were age less than 18 years and a history of diabetes or renal disease.

### Objectives and outcome

Skin AF was assessed in ICU patients and healthy controls at baseline, on 7 consecutive days. Blood markers were assessed at baseline and on 7 consecutive days in ICU patients and only once in healthy controls. Because of limitations in the availability of blood samples, CRP, CML/CEL, LDL dienes and sRAGE were determined for 19, 25, 33 and 29 healthy controls, respectively. Clinical and routine laboratory parameters were assessed at baseline in ICU patients and healthy controls, and on 7 consecutive days in ICU patients. The primary analysis variable was the difference in Skin AF between ICU patients and healthy controls. Serum CML, CEL, LDL conjugated dienes, sRAGE at inclusion, the time course of Skin AF and circulating markers during the first 7 days after inclusion, organ failure, and in-hospital mortality were secondary analysis variables.

### Organ failure

To quantify organ failure, the parameters of the Sequential Organ Failure Assessment (SOFA) score were used: paO2/FiO2 ratio for respiratory function, thrombocytes for hematologic function, bilirubin level for liver function, inotropic dosage for circulatory function, and creatinine level at inclusion and the need for renal replacement therapy for renal function [19].

### AGE accumulation in the skin

The AGE-Reader (DiagnOptics Technologies BV, the Netherlands) and the procedure which was used to measure the Skin AF have been described before [20–22]. The AGE-Reader has been validated against skin biopsy levels in earlier studies in healthy subjects and patients with diabetes or renal disease of the following AGEs: pentosidine, CML and CEL, as well as with collagen-linked-fluorescence, which has been classically used as a standard for AGE accumulation in tissue [23–25]. Repeated measurements in controls and diabetic patients showed an intra-individual Altman error percentage of 5.0% on a single day and 5.9% for seasonal changes [23].

Skin AF was measured on the ventral site of both lower arms separately at each time point; the average of these two measurements was used. Measurements were performed at a skin site without visible vessels, scars, bruises, or other abnormalities. Skin temperature was measured at the site of Skin AF measurement using a sensor which was kept in place for 15 minutes, which is long enough to obtain a stable value. The presence of pitting oedema or haematoma at a distance of less than 15 centimetres from the measurement site and the presence of i.v. catheters in the ipsilateral arm was documented.

Previous studies in stable patients have shown that Skin AF can only be measured reliably if the absorption of excitation and fluorescence light in the skin is limited, which is below a skin reflectance of <12%.[20]. Skin reflectance <12% may occur in persons with dark skin, usually Fitzpatrick class VI or sometimes V. Since the current study was an explorative study and it was not clear whether this threshold holds true for ICU patients, we have chosen to analyse data of all patients, irrespective of their skin reflectance. In healthy controls with a wide age-range we have found previously that Skin AF is clearly and strongly related to skin elasticity (measured with Cutometer®), but not to skin hydration (Corneometer®), colour and erythema (both Mexameter) (data not published). Also, Skin AF does not appear to change directly following haemodialysis and ultrafiltration [26]. These data make it plausible to measure Skin AF reliably in ICU patients who have dynamic hydration and skin perfusion states. To assess the reproducibility, the Coefficient of Variation (CV) was assessed in ICU patients by calculating the right to left arm CV and the day-to-day CV and local sources of error were assessed (skin reflectance, local skin temperature, edema, hematoma, and the presence of intravenous catheters).

### Serum markers

Blood samples were taken at the time of inclusion and in the next 6 days during early morning blood sampling rounds. The blood was allowed to clot at room temperature in dark for 30–60 minutes. The serum was separated by centrifugation (2000g for 10 min at room temperature) and divided in polypropylene tubes. EDTA (1 mg/ml) was added to the samples for LDL dienes determination to prevent auto-oxidation of fatty acids. The samples were frozen immediately and stored at -80°C until analysis.

Serum levels of CML and CEL were measured in the protein fraction by stable-isotope dilution tandem mass spectrometry [27,28]. Briefly, serum proteins were reduced with 100 mM sodium borohydride in 0.2 M sodium borate buffer at pH 9.2 for 2 h. The proteins were precipitated with 20% trichloroacetic acid and hydrolyzed with 6 M HCl overnight at 110°C. HCl was evaporated at 70°C under nitrogen and residues were resolved in 0.5 mM tridecafluoroheptanoic acid. Analysis was performed using a C18 reverse phase column with a linear gradient of acetonitril. CML and CEL were measured using a positive ionization mode with respectively [2H2]-CML or [2H4]-CEL as internal standard. Intra- and inter-assay coefficients of variation were 4.8 and 7.0% for CML, and 5.0 and 9.7% for CEL, respectively. Concentrations of protein-bound CML and CEL were adjusted for total protein levels and expressed as nmol/gr protein.

Serum levels of sRAGE were measured using a commercially available ELISA (R&D systems, Minneapolis, MN, USA) according to the manufacturer’s protocol. The intra- and inter-assay CV were <10%.

The oxidation of LDL in vivo was assessed as the concentration of conjugated dienes in precipitated LDL as previously described [29]. We used nitric acid washed screw-capped glass tubes in all steps of analysis in order to avoid artefacts. Briefly, serum LDL was precipitated with buffered heparin (LEO Pharma A/S (Ballerup, Denmark)). The precipitate was resuspended in 0.10 M phosphate buffered saline, pH 8.0. Cholesterol concentration (detection limit 0.20 mmol/L) was determined with an enzymatic colorimetric method by Konelab 20XT analyzer (Thermo Fisher Scientific, Vantaa, Finland) and the rest of the suspension was used for measuring conjugated dienes. Lipids were extracted from the LDL with the mixture of chloroform:methanol (3:1), evaporated to dryness with a gentle stream of nitrogen, and reconstituted in cyclohexane. The concentrations of conjugated dienes were measured spectrophotometrically at 234 and 300 nm. Absorbance units (difference A234–A300) were converted to molar units using the molar extinction coefficient 2.95 × 10^4^ M^−1^ cm^−1^ for conjugated dienes. The conjugated diene concentration was calculated per cholesterol concentration in LDL (μmol/mmol cholesterol). Interassay variations were 12.7% (*n* = 140, serum pool at conjugated diene level of 8.3 μmol/mmol cholesterol) and 11.9% (*n* = 66, serum pool at conjugated diene level of 8.6 μmol/mmol cholesterol) and the detection limit of conjugated dienes was 10 μmol/L.

### Statistical analysis

Power analysis was based on a small pilot study [30]. It was calculated that 28 patients and 28 controls had to be included to detect a difference in Skin AF of 0.33 au (15%) based on a standard deviation of 0.44 at a significance level of 0.05 with a power of 80%. Taking into account a dropout rate of 35%, we included 45 patients.

Values are expressed as mean + standard deviation, median (interquartile range), or as the geometric mean + standard deviation where applicable. Analyses were performed using Statistical Package for Social Science version 22 (International Business Machines Corp., New York, USA) or Graphpad Prism version 5.0 (Graphpad Software Inc., San Diego, USA). Differences between groups of quantitative variables were analysed by student’s t-test if they were normally distributed. Where appropriate a Welch correction was applied. For non-normally distributed data, the Mann-Whitney U test was used. Qualitative variables were analysed by Pearson’s chi-square test. To compare multiple groups of measurements one-way analysis of variance or the Kruskal-Wallis test were used. Correlations between numeric data were analyzed with the Spearman rank method.

Stepwise linear regression was used to correct for potential confounders that show a univariate correlation with Skin AF at p<0.10 at baseline. Forward conditional logistic regression was used to test whether the difference in Skin AF between patients and controls was independent of confounders that were available, i.e. a history of cardiovascular disease and hypertension. Age and gender were not included in the analysis because groups were matched on these variables. To analyse time dependent associations of repeated measurement during the first 7 days in ICU patients, Generalized Estimating Equation (GEE) analysis was used and data are presented as (geometric) mean with 95% confidence intervals. Only if GEE show time association with p<0.10, additional comparisons at individual time points were assessed using the above mentioned statistics. Survival was estimated by the Kaplan-Meier method, and comparisons were made with the log-rank test. A p-value of 0.05 or less was considered to be statistically significant.

## Results

### Baseline characteristics

Table 1 presents the characteristics of patients and healthy controls. All patients and controls were Caucasian. ICU patients were critically ill, as evidenced by a median Apache II score of 24 at inclusion and an in-hospital mortality rate of 51 percent (3 patients died at the ward, all others at the ICU). The mean SOFA score at inclusion was 11 + 4. Renal replacement therapy, vasopressor therapy, and artificial ventilation were required in 62%, 98% and 96% of cases, respectively. Most patients had sepsis with pneumonia and peritonitis as most important sources.

**Table 1:** Baseline and outcome variables of ICU patients and healthy controls.

		controls	ICU patients	p-value
n =		37	45	
age [years]		59±14	59±15	0.93
Men		25 (68%)	27 (60%)	0.48
cardiovascular disease		0	7 (16%)	0.011*
hypertension		3 (8%)	14 (32%)	0.009**
diagnosis	pneumonia		12 (27%)	
	peritonitis		12 (27%)	
	cholangitis		4 (9%)	
	sepsis, other		7 (15%)	
	pancreatitis		3 (7%)	
	other		7 (15%)	
Apache II score at inclusion			24 (21–29)	
SOFA score at inclusion			11±4	
CRP [mg/L]		1.3 (0.7–2.2)^a^	224 (128–320)	<0.001***
glucose [mmol/L]		4.8 (4.6–5.2)	6.6 (5.6–7.4)	<0.001***
creatinine [μmol/L]		88 (84–97)	189 (128–267)	<0.001***
treatment				
renal replacement therapy			28 (62%)	
vasopressor			44 (98%)	
ventilation			43 (96%)	
days between hospital admission and inclusion			3 (1–14)	
days between ICU admission and inclusion			2 (1–3)	
days between inclusion and death or discharge			23 (6–43)	
length of stay [days]	hospital		37 (15–55)	
	icu		13 (6–31)	
death in hospital			23 (51%)	
skin AF [au]		1.8±0.3	2.7±0.7	<0.001***
serum CML [nmol/gr protein]		29 (25–33) ^a^	27 (20–39)	0.64
serum CEL [nmol/gr protein]		16±3 ^a^	23±10	<0.001***
serum LDL dienes [μmol/mmol cholesterol]		9 (8–11) ^a^	19 (15–23)	<0.001***
serum sRAGE [pg/ml]		1042 (824–1388) ^a^	1547 (998–2496)	0.003**

^a^ Due to limitations in the availability of blood samples, CRP, CML/CEL, LDL dienes and sRAGE were determined for 19, 25, 33 and 29 healthy controls, respectively.

*:p<0.05

**:p<0.01

***: p<0.001

Compared to the healthy controls, partly due to exclusion criteria for controls, significantly more ICU patients had a history of cardiovascular disease or hypertension and higher levels of CRP, glucose, and creatinine at inclusion.

### Reproducibility of Skin AF in ICU patients

No structural difference was observed between Skin AF measured at the left and right arm of individual ICU patients. The right-left arm CV was 13% and the day-to-day CV was 10%. The CVs of skin reflectance were 11.6% and 6.8% respectively

### Influence of local factors on skin AF measurement

Single measurements of Skin AF of the right and left arm correlated moderately with skin reflectance measured at the same site (r = 0.391, p = 0.012 and r = 0.297, p = 0.059 respectively), whereas no relation was observed with local oedema of the arm, hematoma, and whether intravenous catheters were present at the ipsilateral arm. Also, skin temperature did not correlate with Skin AF.

### Comparison of Skin AF between ICU patients and controls

**Fig 1:**
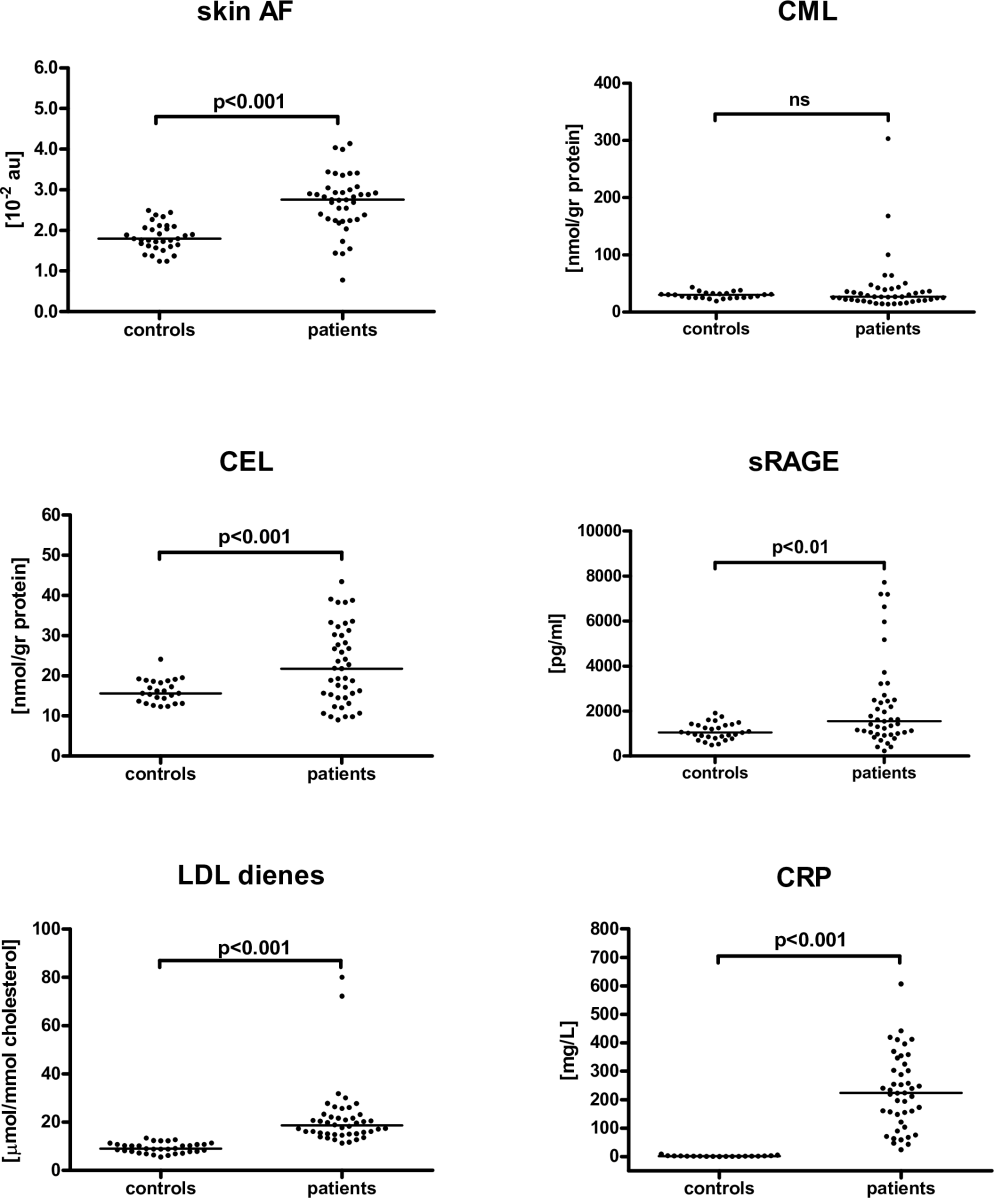
Dot density plot of Skin AF, serum AGEs, sRAGE, LDL dienes and CRP at inclusion, comparing ICU patients with healthy controls.

Skin AF at inclusion, which was defined as the primary analysis variable, was significantly higher in ICU patients compared to healthy controls (Fig 1). This difference remained significant (p = 0.015) after correction for skin reflectance, glucose, creatinine, prior hypertension, or prior cardiovascular disease (p = 0.91).

### Association of skin AF with baseline characteristics and circulating markers in ICU patients

Skin AF measured at baseline, correlated positively with age (r = 0.49; p = 0.001), negatively with LDL dienes (r = -0.36; p = 0.017) and bilirubin levels (r = -0.39; p = 0.008; Table 2), and was significantly higher in smokers (3.1 vs 2.5; p = 0.04). It was not dependent on gender (p = 0.19), did not correlate with the time between hospital or ICU admission and inclusion (r = -0.11; p = 0.46 and r = -0.17; p = 0.27) and tended to be higher in patients on renal replacement therapy (2.8 vs 2.4; p = 0.06) and prior hypertension (2.9 vs 2.5; p = 0.09). Although skin AF correlated negatively with CML (r = -0.33; p = 0.029), this difference was blunted after correction for bilirubin levels. Skin AF did not correlate with baseline CEL, CRP or sRAGE (Table 2). Stepwise, linear regression yielded that of these factors only age was independently associated with Skin AF (R^2^ = 0.43; standardized beta 0.66; p = 0.001).

**Table 2:** Correlations between Skin AF, serum AGEs, sRAGE, LDL dienes, CRP and bilirubin level in critically ill patients at inclusion.

Spearman's rho (p-value)	CML	CEL	CRP	LDL dienes	sRAGE	bilirubin
Skin AF	-0.33 (0.029)*	-0.13 (0.40)	0.07 (0.66)	-0.36 (0.017)*	0.06 (0.69)	-0.39 (<0.01)**
CML		0.71 (<0.001)***	-0.56 (<0.001)***	0.12 (0.43)	0.22 (0.16)	0.66 (<0.001)***
CEL			-0.41 (<0.01)**	0.08 (0.60)	0.22 (0.16)	0.35 (0.025)*
CRP				-0.12 (0.46)	-0.16 (0.32)	-0.27 (0.08)
LDL dienes					-0.07 (0.65)	0.10 (0.54)
sRAGE						0.20 (0.21)

*:p<0.05

**:p<0.01

***: p<0.001

### Correlations between serum AGEs, CRP, LDL dienes, sRAGE and bilirubin (Table 2)

Positive correlations were found between CML and CEL (r = 0.71, p<0.001) and between CML or CEL and bilirubin (r = 0.66, p<0.001 and r = 0.35, p = 0.025). CML and CEL correlated negatively with CRP (r = -0.56, p<0.001 and -0.41, p<0.01).

### Comparison and intercorrelation of serum markers between ICU patients and healthy controls

Serum levels of CEL, sRAGE, LDL dienes, and CRP were significantly higher in ICU patients compared with healthy controls (Fig 1). The median CML did not differ between patients and controls, but an increased variance was observed in patients. The distribution of Skin AF, CRP, CML, CEL, LDL dienes, and sRAGE were the same across the diagnosis categories listed in Table 1 (Kruskal-Wallis Test, p = 0.52, 0.29, 0.08, 0.27, 0.77 and 0.19, respectively). The Apache II score did not correlate with any of the markers, whereas the SOFA score correlated with CML and CEL levels (r = 0.54 (p<0.001) and 0.46 (p = 0.002) respectively). SOFA score did not correlate with Skin AF, sRAGE, LDL dienes and CRP. Analysing the SOFA components revealed a strong correlation between CML and the hepatic score (based on bilirubin level) only (Kruskal-Wallis test, p = 0.001).

### Association of baseline Skin AF and serum markers with disease outcome

**Fig 2:**
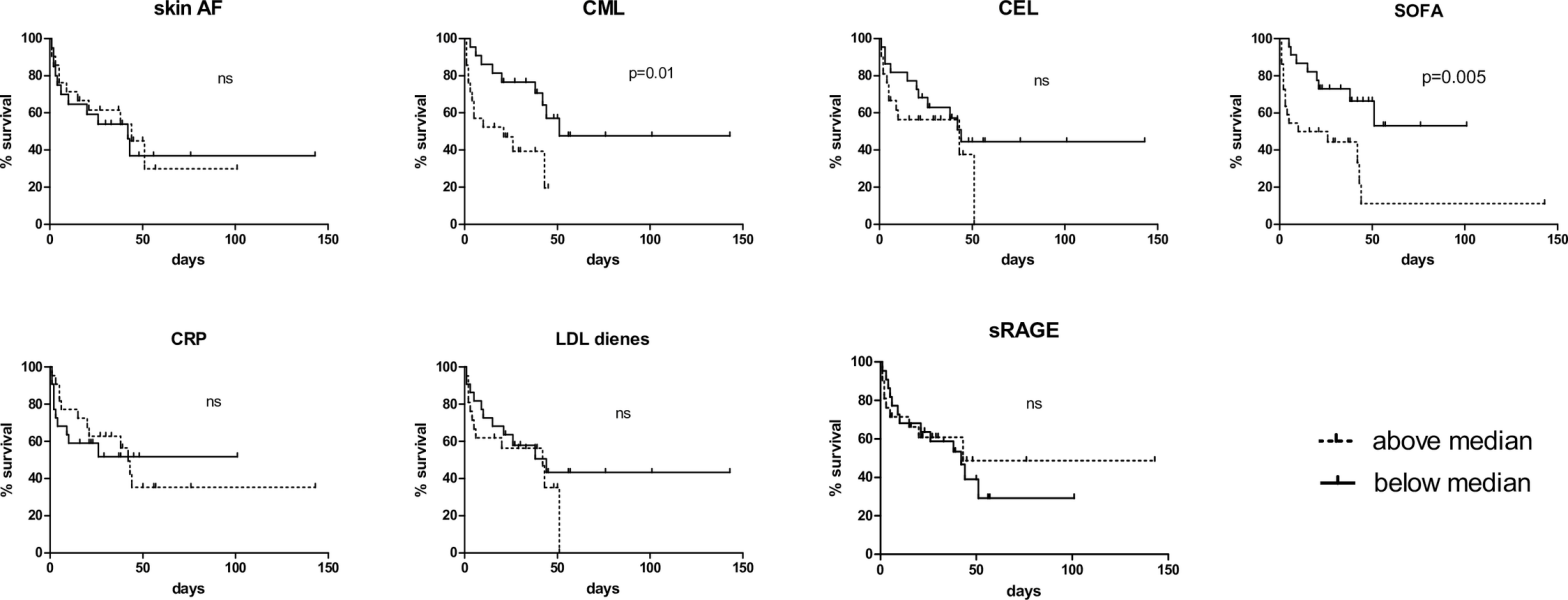
Kaplan-Meier survival curves of Skin AF, serum AGEs, sRAGE, LDL dienes, CRP and the SOFA score. For each variable, the patients were split in two groups, namely those with values above the median and those having values below the median. The two groups were compared by using the log rank test.

Patients who died had a significantly higher SOFA score and were more likely to have been admitted for pneumonia. However, non-survivors did not differ from survivors in age, cardiovascular history, and APACHE II score, nor were there any significant differences in Skin AF, CML, CEL, LDL dienes, and sRAGE. Fig 2 shows survival curves, in which patients with values above the median were compared with those below the median of the variable under consideration. As expected, SOFA score was a positive predictor of hospital mortality. Additionally, CML above the median was positively associated with mortality (Hazard ratio 3.3 (1.3–8.3); p = 0.01). All the other markers determined in this study, i.e. Skin AF, CEL, CRP, LDL dienes and sRAGE, did not predict mortality.

### Repeated measurements in ICU patients

**Fig 3:**
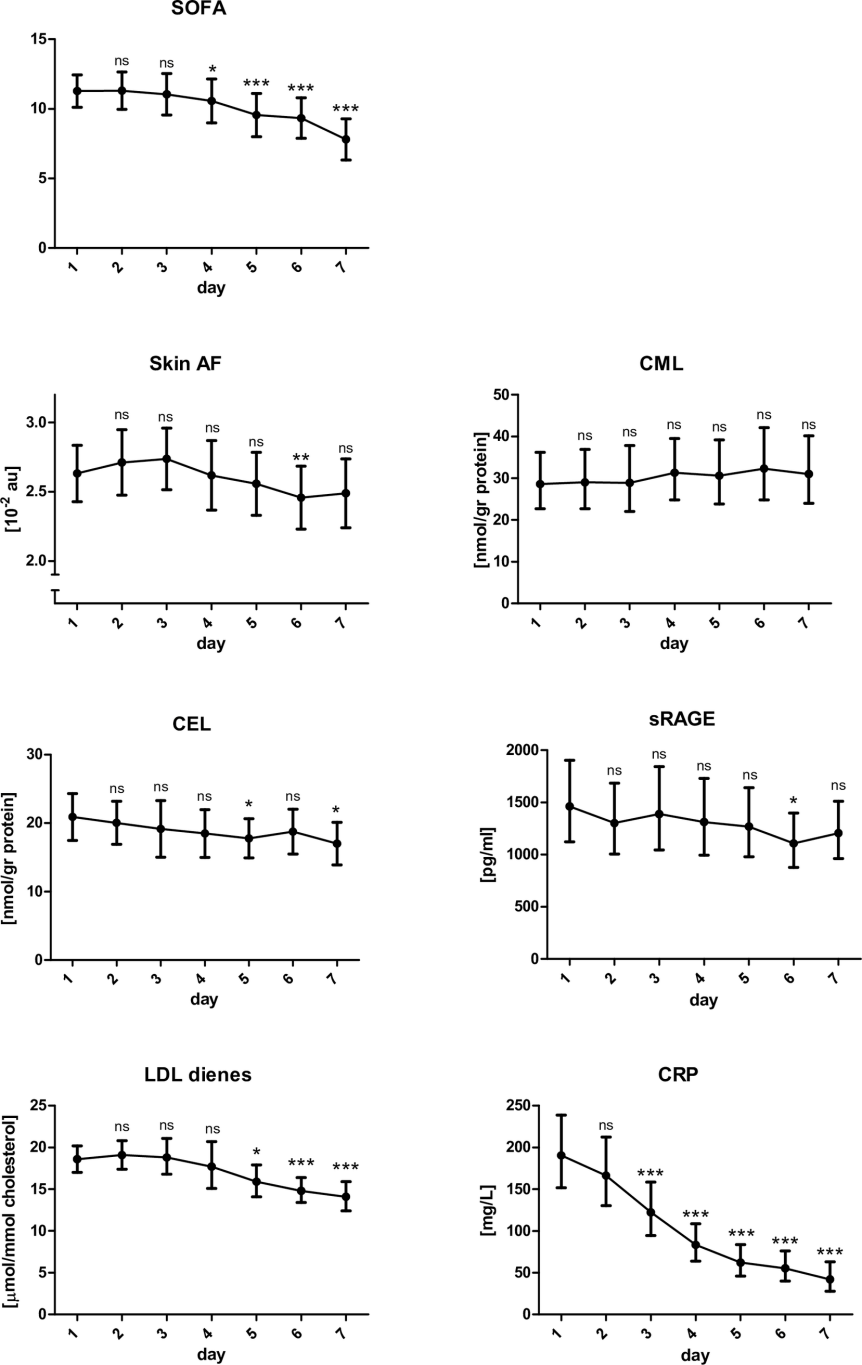
Time course of SOFA score, Skin AF, circulating AGEs (CML and CEL), sRAGE, LDL dienes and CRP of ICU patients during 7 days starting at inclusion. Geometric mean with 95% confidence intervals. The symbols indicate the p-value compared to day 1: ns = non-significant; *:p<0.05; **:p<0.01; ***: p<0.001.Patients who died or were discharged before day 7 were excluded from this analysis. GEE repeated measurement analysis showed that SOFA score (p<0.001), LDL dienes (p<0.001) and CRP (p<0.001) were time related, while Skin AF (p = 0.09), CML (p = 0.55), CEL (p = 0.26) and sRAGE (p = 0.082) were not.

GEE repeated measurement analysis demonstrated small and non-significant time effect on Skin AF (p = 0.09), with only a small decrease in Skin AF from baseline (2.59 (2.37–2.81)) to day 6 (2.42 (2.18–2.66); p = 0.012) and day 7 (2.45 (2.19–2.72); p = 0.086) in those with an ICU length of stay of at least 7 days (Fig 3). Other relevant parameters identified as changing over time were SOFA score (decrease; p<0.001; Fig 3), CRP (decrease; p<0.001; Fig 3), creatinine (decrease; p<0.001), body weight (increase; p<0.001), PaO2/FiO2 ratio (increase; p<0.001), norepinephrine dose (decrease; p<0.001), whereas glucose (p = 0.99) did not change significantly. Of these parameters, CRP (p = 0.014), creatinine (p = 0.026), and body weight (p = 0.015) were significantly associated with Skin AF over time, whereas SOFA (p = 0.113), PaO2/FiO2 ratio (p = 0.91), and norepinephrine dose (p = 0.77) were not. In multivariate analysis, only CRP and body weight were independently associated with Skin AF over time. The change in Skin AF from baseline to day 7 did not differ between those who died in the hospital after surviving the first week and those who survived until discharge from the hospital (change in Skin AF: -0.16±0.25 vs -0.12±0.46; p = 0.70).

### Repeated measurements of blood markers and their relation with Skin AF and disease severity

In those ICU patients who participated in the study for at least 7 days, CML (p = 0.55), CEL (p = 0.26), and sRAGE (p = 0.082) did not change significantly over time. LDL dienes showed a significant decline starting at day 5 compared to baseline (p<0.001). In multivariate analysis, the change in CEL (p = 0.46), sRAGE (p = 0.89), and LDL dienes (p = 0.41) did not correlate with the change in Skin AF. CML was negatively associated with Skin AF (p = 0.028). Skin AF as well as CML were both also strongly associated with bilirubin levels over time, with Skin AF showing a negative association (p = 0.023) and CML a positive (p<0.001). The association of Skin AF with CML was blunted after correction for the change in bilirubin levels.

## Discussion

In the current pilot study we explored the role of the AGE-RAGE axis in patients admitted to ICU for critical illness. We have demonstrated that Skin AF, as a marker of chronic AGE accumulation, can be measured in patients admitted to the ICU with a reasonable reproducibility, but that several method and patient related confounding factors should be taken into account, limiting far-reaching conclusions. This study shows that Skin AF is elevated in critically ill patients compared to age-matched healthy controls, for which the study was primary powered. Skin AF measured at baseline was independently associated with age only and not with factors indicating acute illness such as SOFA score or CRP or circulating levels of markers of AGEs and oxidative stress. Skin AF showed only a small decreasing non-significant trend during the first 7 days after inclusion and was not associated with in-hospital mortality. At baseline, circulating AGEs (CEL), sRAGE, and LDL dienes were significantly higher in ICU patients compared to healthy controls. Although CML was not elevated, both CEL and CML were clearly associated with markers for disease severity, including the well validated SOFA score. Furthermore, although CRP and LDL dienes as markers for inflammation and oxidative stress clearly decreased over time, all markers for AGEs appeared to remain stable and did not parallel these changing markers. Finally, CML was positively and prospectively associated with in-hospital mortality, while the other markers of the AGE-RAGE axis were not.

Because of several potential confounding factors at the ICU, we performed additional reproducibility tests, showing that the CV between the right and the left arm as well as the day-to-day CV were acceptable but higher than commonly seen in stable patients in previous studies. We previously found that, in healthy subjects, skin AF is not influenced by skin colour and hydration. In the current ICU population, we identified a negative association with skin reflectance, indicating that the more light is absorbed, the lower the skin AF. This effect was small and inconsistent between both arms. Also, some effects of local skin temperature and haematoma were present, but these can be accounted for when performing the measurement. An interesting observation was the strong negative association with plasma bilirubin levels. This may have been caused by absorption of autofluorescence light in the skin as bilirubin is known to strongly absorb light of a wave length of around 460 nm, which is in the spectrum of the autofluorescence measured with our method. These factors may have blurred the results and may have masked subtle associations in the population.

Our baseline data on skin AF are in line with a previous study by Greven and others, who showed remarkably higher age-adjusted Skin AF levels in patients with sepsis compared with controls but no relation with severity of disease [16]. It remains unclear whether or not Skin AF has increased during and as a result of the current acute illness. It can be hypothesized that Skin AF was already higher prior to the current illness and reflects a higher susceptibility for critical illness as a derivative of biological aging and decreased general health. This is substantiated by our observation that Skin AF did not change during admission, with a day-to-day CV of only 10%. Since the level of Skin AF observed in the patients from our study is markedly higher than we measured in patients with chronic diseases [31]), at least an additional effect of the acute illness is suggested. This may be explained by the differences in dynamics between tissue and circulating AGEs. Tissue AGEs may be rapidly formed, but their turnover is very slow and dependent on the half-life of collagen and other long lived proteins. Although circulating AGEs such as CML are rapidly formed or derived from exogenous sources (food, smoking, certain drugs), but are also cleared very rapidly by the kidneys or catabolised in concurrence with circulating proteins [7–12], these levels did not change during the current study. This may be explained by the presence of renal failure in most ICU patients. This may also explain the lack of correlation between serum AGEs and skin AF, which was previously also observed in chronic diseases such as diabetes and renal failure [32]. Moreover, it has been shown that AGEs (especially methylglyoxal (MGO)) may suppress myeloid and T-cell immune function and increase susceptibility to infections, referred to as “immune glycation damage” [33], thereby preselecting patients with higher a priori Skin AF levels.

It has been hypothesized that AGEs are causally involved in the exaggerated inflammatory response by engagement of the multiligand receptor for AGEs (RAGE) [13], as supported by rodent studies [14]. The results from our study are mixed and cannot substantiate this hypothesis. A recent study by Andrades et al. found an inverse association between blood CML/CEL levels and severity of sepsis and renal function [34]. The authors suggest that AGEs participate in early stages of renal dysfunction by increasing permeability resulting in proteinuria. However, if renal failure is already present, a wide base of literature suggests that circulating AGEs would be increased [35–37]. Also, loss of AGEs due to proteinuria could not be the explanation for the lower levels, because they quantified AGE levels relative to grams of protein in the same way as we did in the current study. Therefore, it remains unclear how to explain these apparently conflicting data and this underlines the complex nature of the AGE/RAGE axis.

Although an inverse association between bilirubin and AGE levels was previously found in subjects with Gilbert syndrome [38], in line with the hypothesis that bilirubin may have antioxidative properties [39], we observed the opposite. A strong positive correlation between bilirubin levels and CML was documented, which persisted after exclusion of extreme outliers. This suggests a role of the liver in the production or breakdown of CML [40] and that it is not bilirubin per se, but the degree of liver failure that determines the levels of AGEs.

With respect to sRAGE, our results are in accordance with other studies which show increased levels of sRAGE in critically ill patients with sepsis and acute lung injury [41–43]. However, we were not able to confirm the positive correlations of sRAGE level with mortality, lung injury (paO2/FiO2 ratio), or renal replacement therapy that have been found by others [41,44–46]. Brodska et al. studied a cohort of 54 critically ill septic patients and observed a significant correlation between sRAGE and CRP on day 1, while they didn’t find a correlation between these two parameters on day 3 [44]. This shows that correlations may change in the course of the disease, possibly reflecting a changing role, for example from being a by-product of apoptosis to getting a direct beneficial role by acting as a decoy receptor for RAGE ligands such as AGEs, as has been suggested by others. Therefore the timing of measuring sRAGE in the course of the disease may be essential for the interpretation of the results.

A remarkable finding in our study was the strong elevation of plasma CEL levels, whereas the levels of CML were not increased. This may be explained by the fact that CEL and CML are derived from different sources: CML is derived from glyoxal and CEL from MGO. MGO can be detoxified by glyoxalase 1 (GLO-1), which is an enzyme that has been shown to exhibit reduced activity after stimulation with inflammatory mediators. Hence, resulting in increased accumulation of MGO-derived AGEs such as CEL [28]. Unexpectedly, CRP correlated inversely with serum levels of CML and CEL. This correlation was found with and without correction for total serum protein. In chronic settings, such as in patients with diabetes, some authors found a positive correlation between CRP and blood AGEs [35,47], while others did not [48,49]. An inverse correlation between CRP and CML in blood has also been found in obesity [50], and it has been hypothesized that inflammation may result in trapping of CML in tissue.

In the heterogeneous ICU population, the relation between CRP and AGEs formation may be more complex, as CRP may predict multiple organ failure and poor outcome early in the course of disease [51], while it may not be of prognostic significance in patients who already have multiple organ failure [52], as in our study. It is difficult to speculate on the course of AGEs during acute critical illness, because the relationship between CRP and AGEs may change during the course of the disease and we did not document the course of AGEs prior to developing multiple organ failure.

Although we found CML above the median to be associated with an increased mortality, which is in line with our hypothesis of a detrimental role of oxidative stress and the AGE-RAGE axis in critical illness, we are reticent to draw far reaching conclusions. First, the other AGE we studied, CEL, appeared not to be associated with increased mortality, nor did the LDL dienes as markers of lipid peroxidative stress. This may suggest a random effect. Furthermore, literature on this relation is conflicting. In hemodialysis patients, increased CML was associated with mortality in one study [53], while it was found to be protective in another study [54]. Andrades et al. also found that high levels of a combined ELISA assay for CML/CEL were associated with a decreasing SOFA score and a better survival in patients with sepsis [34]. However, this study included patients with a broader range of sepsis severity, with 20% of the patients having sepsis without organ failure. This may be essential because patients were included within 12 hours after sepsis diagnosis, while we included patients when they already had multiple organ failure. This again underlines the importance of the timing of sampling during the course of the disease.

### Limitations

Major limitations of this study are the relatively small sample size and the heterogeneity of the ICU population. Due to the small sample size, small interactions could have been overlooked. Additionally, the magnitude of repeated assessment of biomarkers, could have introduced coincidental findings. However, we have overcome this limitation be performing GEE analysis for each biomarker. Our control group was too healthy to account for confounding factors such as comorbidity and the contribution of the infection per se. Partly, we have overcome this limitation by excluding patients with previous diabetes of renal failure. As AGEs and LDL dienes are protein and lipid bound respectively, changes in protein and lipids during the catabolic state of severe illness may have influenced the results. We have tried to bypass this by correcting for total protein and cholesterol respectively. Also, infusion fluids used on the ICU may contain amounts of glucose degradation products, which are precursors of AGEs, especially MGO-derived AGEs such as CEL [55,56].

## Conclusions

With the current study we underscore the complexity of AGE-RAGE axis in critical illness. To the best of our knowledge, this is the first study to sequentially measure Skin AF and its potential confounders, circulating AGEs, and oxidative stress markers in critically ill patients. Although CRP and the oxidative stress marker LDL-dienes do clearly decrease during the course of ICU admission, circulating AGEs as well as Skin AF remain fairly stable, and hence their role as a disease marker in ICU patients is questionable. Since all markers appeared to be elevated in critically ill patients and circulating AGEs seemed to be associated with disease severity and, potentially, outcome, the role of the AGE-RAGE axis deserves further study. To unravel the course and relevance of AGE formation in critical illness, patients prone to develop a systemic inflammatory response should be included early after, or even before the trigger. The results from the current study will help in designing such future studies.

## Supporting Information

S1 File xls Dataset of this study. (XLS)
